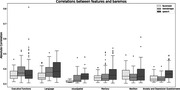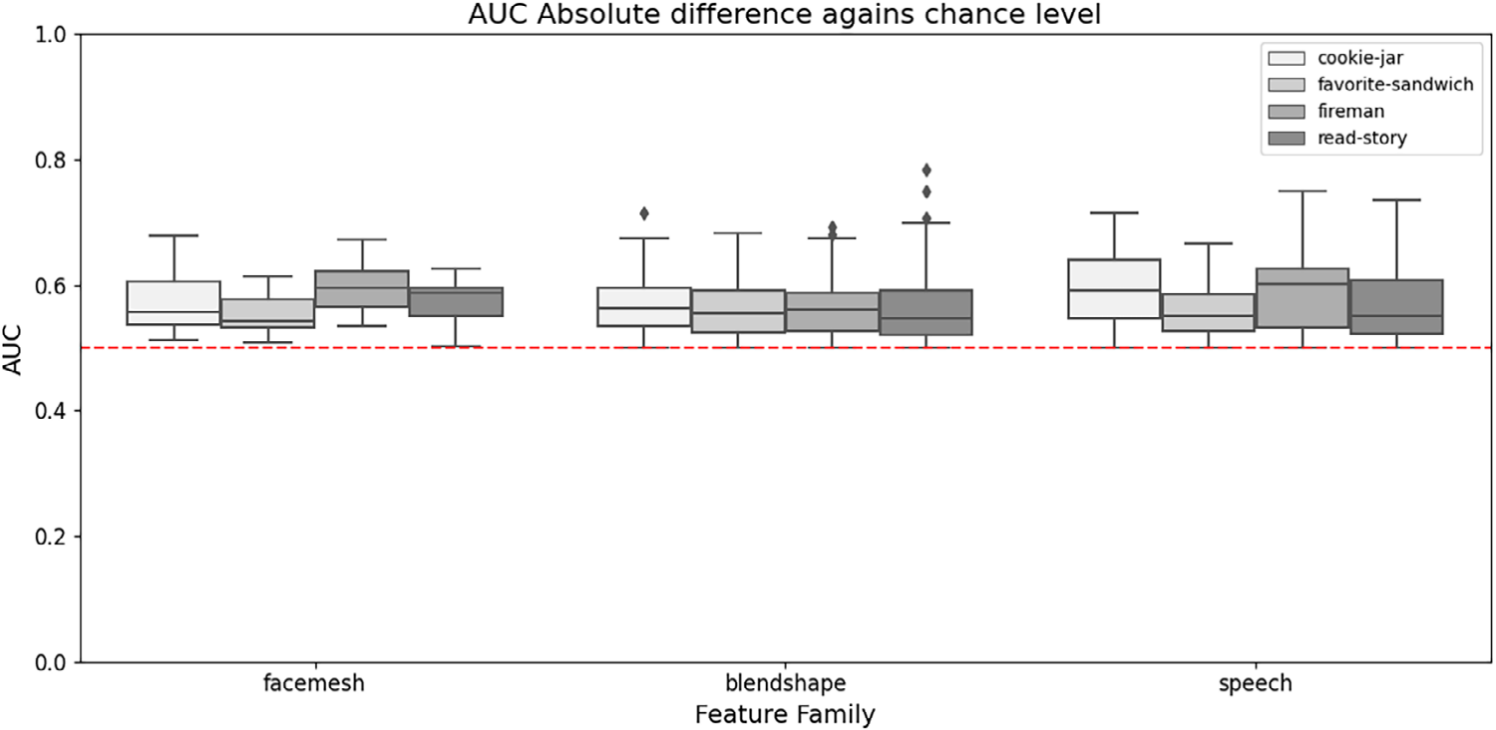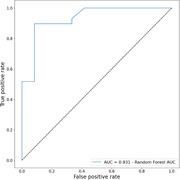# Multi‐modal AI screening for MCI and Alzheimer's Disease: results from an Argentine Cohort

**DOI:** 10.1002/alz.087333

**Published:** 2025-01-09

**Authors:** Laouen Belloli, Greta Keller, Agostina Carello, Lara Gauder, Gustavo Juantorena, Nicolas Corvalan, Ricardo Allegri, Lucia Crivelli, Diego Fernandez Slezak

**Affiliations:** ^1^ Universidad de Buenos Aires, Ciudad Autonoma de Buenos Aires, CABA Argentina; ^2^ Fleni, Buenos Aires, CABA Argentina; ^3^ Fleni, Buenos Aires, Buenos Aires Argentina; ^4^ Universidad de Buenos Aires, CABA, CABA Argentina

## Abstract

**Background:**

Early detection of Mild Cognitive Impairment (MCI) is crucial for effective prevention. Traditional methods like expert judgment, clinical evaluations, and manual linguistic analyses are now complemented by Artificial Intelligence (AI). AI offers new avenues for identifying linguistic, facial, and acoustic markers of MCI. The exploration of these markers for MCI research is just beginning, especially in regions like Argentina. The effectiveness of AI‐methodologies hinges on local data calibration and validation prior to deployment. This study focuses on designing AI‐based neuropsychological instruments to differentiate healthy individuals from MCI subjects, emphasizing local data calibration and validation.

**Method:**

We recruited 42 participants (30/12 healthy/MCI according to Petersen's criteria, 2016) aged 60 to 89 (mean ± SD: 70.95 ± 6.8) in Fleni, Argentina. During the assessment, which included neuropsychological tests (UDS‐3), participants were video and audio recorded while performing a language task on a web platform. They were required to describe two target images ('Cookie Theft' and 'Firefighter‐Oasis.') and two tasks without any target image (description of their favorite sandwich and reading a story). Markers were extracted from 5 modalities: Speech (automated speech transcription), Acoustics (audio), Face mesh (video), Blend shapes (video) and Emotion recognition (video and audio).

**Result:**

Unimodal analysis was performed to study shared information between proposed AI‐markers and traditional neurocognitive tests. Speech markers were correlated with language and memory, and overall, we obtained a total of 204 significantly correlated AI‐markers to traditional tests of a total of 432 (47%). Univariate AUC for MCI diagnosis was measured for all markers obtaining an average above chance performance of (0.57 ± 0.062). Finally, a multivariate random forest classifier was used to extract the performance of the modalities altogether, an AUC of 0.91

**Conclusion:**

The results of this preliminary study show the validity of the AI algorithms applied to the local argentine population. Validation of algorithms using data in Spanish language and with Latin American population is a key element for de dissemination and the diversification of AI tools. The next step of this research is to include more patients with MCI to gain a comprehensive understanding of differential communicational patterns.